# Comparative Pathogenicity of Three Strains of Infectious Bursal Disease Virus Closely Related to Poultry Industry

**DOI:** 10.3390/v15061257

**Published:** 2023-05-26

**Authors:** Kailin Li, Xinxin Niu, Nan Jiang, Wenying Zhang, Guodong Wang, Kai Li, Mengmeng Huang, Yulong Gao, Xiaole Qi, Xiaomei Wang

**Affiliations:** 1 Avian Immunosuppressive Diseases Division, State Key Laboratory for Animal Disease Control and Prevention, Harbin Veterinary Research Institute, The Chinese Academy of Agricultural Sciences, Harbin 150069, China; mqg_2007@126.com (K.L.); nqct17@163.com (X.N.); jiangnan3596@163.com (N.J.); zhangwenying0402@163.com (W.Z.); setback1231@163.com (G.W.); likai01@caas.cn (K.L.); huangmm1017@163.com (M.H.); gaoyulong@caas.cn (Y.G.); 2 World Organization for Animal Health (WOAH) Reference Laboratory for Infectious Bursal Disease, Harbin Veterinary Research Institute, The Chinese Academy of Agricultural Sciences, Harbin 150069, China

**Keywords:** infectious bursal disease virus, very virulent IBDV, novel variant IBDV, attenuated IBDV, pathogenicity

## Abstract

Infectious bursal disease (IBD) is an acute, highly contagious, immunosuppressive, and fatal infectious disease of young chickens caused by infectious bursal disease virus (IBDV). Since 2017, a new trend has been discovered in the IBDV epidemic, with very virulent IBDV (vvIBDV) and novel variant IBDV (nVarIBDV) becoming the two current dominant strains in East Asia including China. In this study, we compared the biological characteristics of the vvIBDV (HLJ0504 strain), nVarIBDV (SHG19 strain), and attenuated IBDV (attIBDV, Gt strain) using specific-pathogen-free (SPF) chicken infection model. The results showed that vvIBDV distributed in multiple tissues, replicated the fastest in lymphoid organs such as bursa of Fabricius, induced significant viremia and virus excretion, and is the most pathogenic virus with a mortality of more than 80%. The nVarIBDV had a weaker replication capability and did not kill the chickens but caused severe damage to the central immune organ bursa of Fabricius and B lymphocytes and induced significant viremia and virus excretion. The attIBDV strain was found not to be pathogenic. Further studies preliminarily suggested that the expression level of inflammatory factors triggered by HLJ0504 was the highest, followed by the SHG19 group. This study is the first to systematically compare the pathogenic characteristics of three IBDVs closely related to poultry industry from the perspectives of clinical signs, micro-pathology, virus replication, and distribution. It is of great importance to obtain an extensive knowledge of epidemiology, pathogenicity, and comprehensive prevention, and control of various IBDV strains.

## 1. Introduction

Infectious bursal disease (IBD) is an acute, highly contagious, and fatal infectious disease of chickens characterized by damage to the central immune organ bursa of Fabricius and its B lymphocytes. IBD causes immune dysfunction in chickens and interferes with the effectiveness of various vaccines to generate immunity [1]. The disease mainly affects young chickens with a morbidity of almost 100%, and the very virulent strains result in higher mortality. IBD has been identified as a disease that has significant socioeconomic impacts.

Infectious bursal disease virus (IBDV), the pathogen of IBD, is a double-stranded RNA virus without envelop and belongs to the genus *Avibirnavirus* in the family *Birnaviridae*. There are two serotypes of IBDV, and only the serotype I virus is pathogenic to chickens. Based on antigenicity and pathogenicity, IBDV serotype I has been classified into classic strains, variant strains, very virulent strains, and attenuated strains used for vaccination. Classic strains of IBDV first appeared in the United States in the late 1950s, in which the infection caused bursal inflammation and severe lymphocytic necrosis, resulting in immunosuppression and a low mortality. In the mid-1980s, variant strains of IBDV emerged in North America, which could evade the immune protection of vaccines against the classic strains. In the late 1980s, very virulent IBDV (vvIBDV) strains suddenly appeared in Europe with a mortality of over 60% and quickly spread throughout the world [2]. For nearly 30 years, vvIBDV strains have caused serious economic losses to the poultry industry worldwide, including China. Wild strains have been attenuated through blind-passage and prepared as vaccines to prevent and control vvIBDV infections [3].

Since 2017, a novel IBDV variant strain (nVarIBDV), different from the earlier variant strain identified in North American, has appeared in China [4] and has become widely prevalent in immunized flocks. Subsequently, the spread of nVarIBDV has been reported in Japan [5], Korea [6], and Malaysia [7]. The nVarIBDV can evade the immune protection induced by vvIBDV vaccine [8,9]. In China, the current prevalence of IBDV has shown a new trend, with the dominant epidemic strain changing from a single vvIBDV to a co-existence of vvIBDV and nVarIBDV [10,11,12,13,14]. In this study, we systematically compared the pathogenic characteristics of three strains of IBDV, including vvIBDV, nVarIBDV, and attenuated strains, from the perspectives of clinical signs, micro-pathology, virus replication, and inflammation-related genes expression. It laid a solid foundation for a comprehensive understanding of the infection characteristics of IBDV with different virulence and for in-depth research on the pathogenic mechanisms of IBD.

## 2. Materials and Methods

### 2.1. Viruses

The representative strains of vvIBDV (HLJ0504) [13] and nVarIBDV (SHG19) [4] were isolated, identified, and conserved by the Avian Immunosuppressive Disease Division at Harbin Veterinary Research Institute (HVRI), Chinese Academy of Agricultural Sciences (CAAS) (referred to as “our laboratory” in the following sections). The attenuated IBDV (attIBDV) strain, Gt, was obtained by attenuating the vvIBDV strain with blind-passage in our laboratory [3]. The TCID_50_ of the viruses was determined with DT40 cells as described previously [10].

### 2.2. Experimental Animals

Specific-pathogen-free (SPF) young chickens were purchased from the National Poultry Laboratory Animal Resource Center and kept in the negative pressure isolators in the Experimental Animal Center of HVRI of the CAAS. The animal study was approved by the Experimental Animal Ethics Committee of HVRI of the CAAS.

### 2.3. Experimental Animals Grouping and Infections

The 21-day-old SPF chickens were randomly divided into four groups: the vvIBDV (HLJ0504), nVarIBDV (SHG19), attIBDV (Gt), and control groups (Mock), with 55 chickens in each group. Virus infection was achieved by the application of eye and nasal drops at a dose of 100 TCID_50_/chicken. The Mock group was treated with 200 μL phosphate buffer solution (PBS) in the same way. The experimental chickens were clinically observed and regularly sampled for testing for a period of 14 days post- infection.

### 2.4. Mean Symptomatic Index (MSI) and Fatality

For the comparison of mortality of different strains, five chickens were randomly selected from each infected group and individually labeled. The clinical signs and deaths in these chickens were monitored daily, and MSIs were determined. MSI has four scoring levels [15]: 0, no clinical signs; 1, showing signs such as depression and ruffled feathers only at rest and returning to normal when exposed to external stimuli with normal motility; 2, showing significant clinical signs even when exposed to external stimuli with reduced motility; 3, exhibiting extremely severe clinical signs with prostration or death.

### 2.5. Autopsy

Five chickens in each group were randomly selected for autopsy at 12 h, 24 h, 36 h, 48 h, 60 h, 3 d, 4 d, 5 d, 6 d, 7 d, and 14 d post-infection (p.i.) to determine lesions in each organ. The lymphoid organs (bursa, spleen, thymus, and caecal tonsil) and non-lymphoid organs (kidney, heart, liver, and lung) were harvested from each chicken. PBS was added to a part of each organ in a 1 mL/g tissue ratio. The tissue was broken by oscillation and freeze-thawed three times to make a homogenate, which was stored at −80 °C for further extraction of RNA and fluorescence-based real-time quantitative reverse transcription PCR (qRT-PCR) detection. The remaining part of each organ was fixed in formalin and kept for sectioning to generate samples for pathological examination. Two bursal samples were randomly selected from each group of dissected chickens and placed in a special fixative solution. These samples were then set aside for IBDV electron microscopy (EM) observation. Additionally, the whole blood with anti-coagulants and serum from each chicken before dissection were collected for viral load detection and antibody detection with ELISA, respectively. Furthermore, cloacal swabs were obtained and stored in 800 µL of PBS at −80 °C for subsequent RNA extraction and qRT-PCR detection.

### 2.6. Organ-to-Body Weight Ratio

At each autopsy, the weights of the whole body, bursa, and spleen of each chicken were recorded, and the bursa weight/body weight ratio (B/BW) and spleen weight/body weight ratio (S/BW) were calculated. B/BW = (bursa weight/body weight) × 1000. S/BW = (spleen weight/body weight) × 1000 [16].

### 2.7. Microscopic Histopathological Observations

Tissue samples fixed with formalin were sectioned, stained by hematoxylin–eosin (H&E), and examined for histopathological features by Pathology Research Division at HVRI.

### 2.8. EM Observation

Fixed bursal samples were sectioned, negatively stained, and observed with electron microscopy by Electron Microscopy Research Division at HVRI.

### 2.9. Detection of Viral RNA in the Tissues

According to the manufacturer’s instructions, RNA was extracted from the prepared tissue homogenates with TRIzol reagent (Invitrogen), and cDNA was synthesized by cDNA first strand synthesis kit (BioRT). The genomic copy number of IBDV was assessed with Premix Ex Taq (TAKARA) on a qRT-PCR instrument (ABI QuantStudio5), and the viral gene copy numbers in tissues and blood were calculated and presented as copies of IBDV RNA/28S rRNA × 10^6^ cells as described previously [17]. The viral gene copy numbers determined in cloacal swabs were presented as copies of IBDV RNA/100 μL. The primers and probes (Table 1) for IBDV genome RNA [18] and chicken 28S rRNA [19] were synthesized by Thermo Fisher Scientific. 

### 2.10. Detection of the Expression Level of Inflammation-Related Genes

DT40 cells were infected with vvIBDV HLJ0504 strain, nVarIBDV SHG19 strain, and attenuated Gt strain. The infection dose was 1 × 10^4^ viral RNA copies/1 × 10^6^ cells. At 24, 36, 48, 60, and 72 h post-infection, the expression level of inflammation-related genes were detected by the relative quantification method of qRT-PCR. The primers used (Table 1) were newly designed and synthesized by Thermo Fisher Scientific.

### 2.11. Serum Antibody Detection

Serum antibodies were detected using an ELISA IBD antibody kit (IDEXX) according to the manufacturer’s instruction.

### 2.12. Statistical Analysis

Data obtained from this study were analyzed using GraphPad Prism 8.0 software. Moreover, the *t*-test and one-way ANOVA were performed to evaluate the differences between groups. Differences with *p* values less than 0.05 were considered statistically significant.

## 3. Results

### 3.1. Clinical Symptoms of IBD

**Figure 1:**
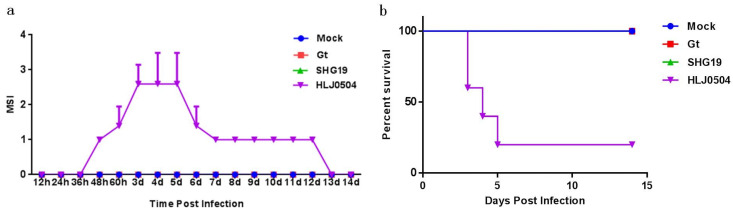
Clinical symptoms of IBD. (**a**) The mean symptomatic index (MSI) of HLJ0504 group. (**b**) The survival curve of chickens infected with different IBDV strains.

Observation records showed that chickens in the HLJ0504 group showed increasingly obvious clinical symptoms at 48 h p.i., mainly including mental depression, decreased appetite, preference for lying down, decreased response to external stimuli, and some chickens excreting loose white stools. Death started to appear in the experimental animals from 3 d p.i., followed by a gradual improvement in the conditions of the chickens that developed tolerance. The MSI curve also showed similar characteristics, with the MSI reaching its highest value at 3–5 d p.i. and then gradually decreasing (Figure 1a). In the chickens dedicated to the analysis of the mortality of the HLJ0504 group, two, one, and one chickens died at 3 d p.i., 4 d p.i., and 5 d p.i., respectively, with a final fatality rate of 80% (4/5) (Figure 1b). Similar to the mock group, neither the SHG19 nor the Gt group had appearance signs or death (MSI = 0). 

### 3.2. Gross Lesions of the Bursa

**Figure 2:**
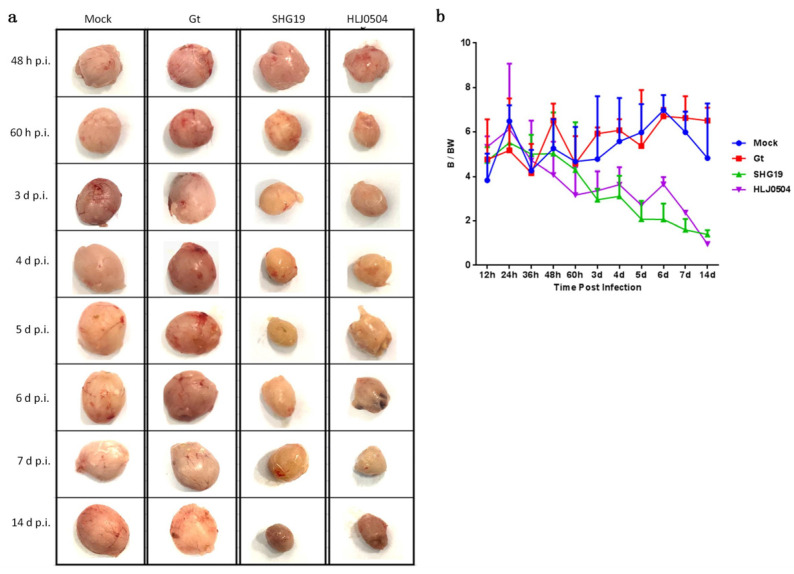
Gross lesions of the bursa. (**a**) The appearance of the bursa in each group from 48 h p.i to 14 d p.i. (**b**) Bursa to body weight ratio (B/BW). The B/BW ratios for each group at each time point were averaged over five chickens, except for the HLJ0504 group at 5 d p.i. to 7 d p.i. (*n* = 3) and 14 d p.i. (*n* = 1).

For the HLJ0504 group, the bursa did not show lesions on appearance from 12 h p.i. to 24 h p.i. At 36 h p.i., 40% (2/5) of the bursas had the appearance indicating caseous necrosis, and at 48 h p.i., 40% (2/5) of the bursas were pale, atrophic, and produced inflammatory mucus. From 60 h p.i. to 14 d p.i., all dissected chickens had a bursa with pale or earthy yellow, atrophic appearance, and exudation of inflammatory mucus. For the SHG19 group, the first visible lesion in bursa was observed at 48 h p.i., with 20% (1/5) of the bursas being pale and exuding inflammatory mucus. At 60 h p.i., 60% (3/5) of the bursas appeared grayish or earthy yellow and exuded inflammatory mucus, and one of the chickens had an atrophied bursa. From 72 h p.i. to 14 d p.i., all dissected chickens had pathological changes in their bursas including atrophy. For the Gt and Mock groups, the appearance, color, and size of the bursa remained normal throughout the test period (Figure 2a).

The dynamic curve of the B/BW ratio showed a similar trend of pathological changes of the bursa to those observed on dissection (Figure 2b). Compared to the Mock group, the atrophy of the bursa was observed in some chickens at 48 h p.i., and all chickens showed atrophic bursa after 60 h p.i with a rapid decrease in the B/BW ratio in the HLJ0504 group. The B/BW ratio reached the lowest at 14 d p.i. In the SHG19 group, some chickens showed atrophic bursa at 60 h p.i., and all had bursal atrophy after 72 h p.i., which deteriorated with time. The B/BW ratios of the Gt group were not significantly different from those of the Mock group during the test period.

### 3.3. Histopathological Lesions of the Bursa

**Figure 3:**
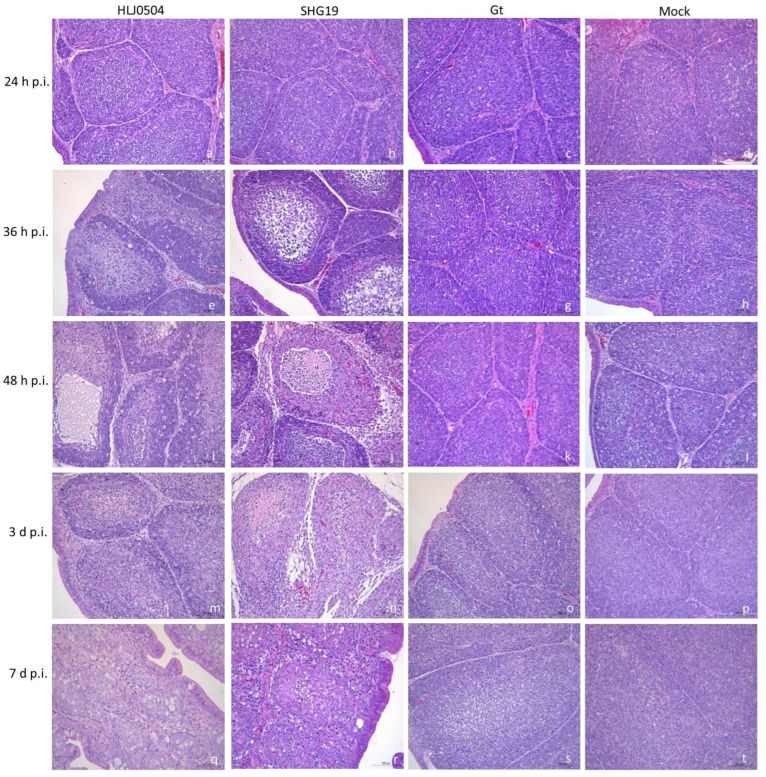
Histopathological changes of the bursa of group HLJ0504, SHG19, Gt, and Mock at 24 h p.i. (**a**–**d**), 36 h p.i. (**e**–**h**), 48 h p.i. (**i**–**l**), 3 d p.i. (**m**–**p**), and 7d p.i. (**q**–**t**).

Compared to the Mock group, typical histopathological changes were observed in the bursa in the HLJ0504 and SHG19 groups, while no pathological changes were found in the Gt group (Figure 3). For the HLJ0504 group, at 36 h p.i., the bursa with gross lesions showed partial follicular atrophy with a significantly decreased number of medullary lymphocytes. At 48 h p.i., in addition to follicular atrophy and a large reduction in lymphocytes due to necrosis, a locally vacuolated medulla with protein-like exudation was observed. After 60 h p.i., a significant infiltration of heterophils and interstitial hyperplasia was observed. Histopathological damage to the bursa remained severe even at 14 d p.i. For the SHG19 group, histopathological changes in the bursa also appeared at 36 h p.i. at the earliest with a remarkable reduction in medulla lymphocytes in some follicles. From 48 h p.i. until 14 d p.i., follicular atrophy progressively deteriorated, with a decrease in excessive necrosis of lymphocytes, notable heterophil infiltration, and interstitial hyperplasia.

### 3.4. Lesions of Other Organs

**Figure 4:**
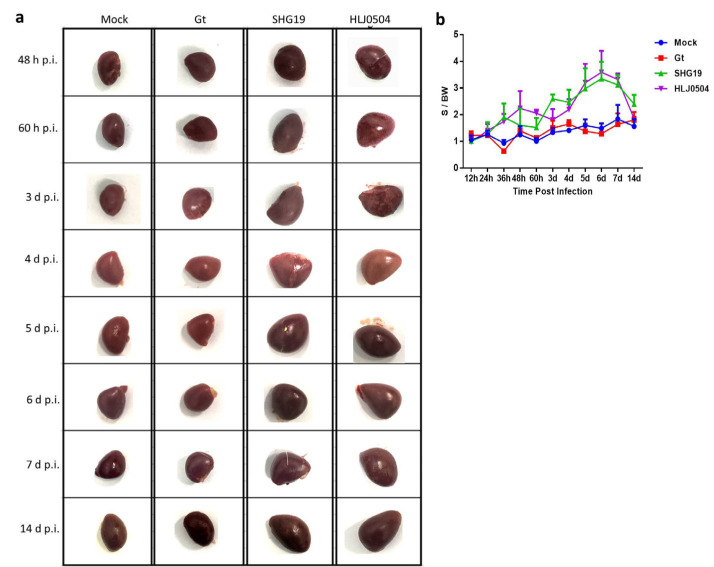
Gross lesions of the spleen. (**a**) The appearance of the spleen in each group from 48 h p.i–14 d p.i. (**b**) Spleen-to-body weight ratio (S/BW). The S/BW ratios for each group at each time point were averaged over 5 chickens, except for the HLJ0504 group at 5 d p.i. to 7 d p.i. (*n* = 3) and 14 d p.i. (*n* = 1).

Spleen: Compared to the Mock group, for the HLJ0504 group, splenomegaly was observed at 36 h p.i. and then became more pronounced over time, and the S/BW ratio reached its peak at 6 d p.i. and then began to decrease. For the SHG19 group, the dynamic changes in the splenomegaly were similar to those of the HLJ0504 group, with the splenomegaly beginning at 36 h p.i., being most severe at 6 d p.i., and remaining significant at 14 d p.i. The chickens in the Gt group had normal spleens, which were not significantly different from the Mock group (Figure 4).

**Figure 5:**
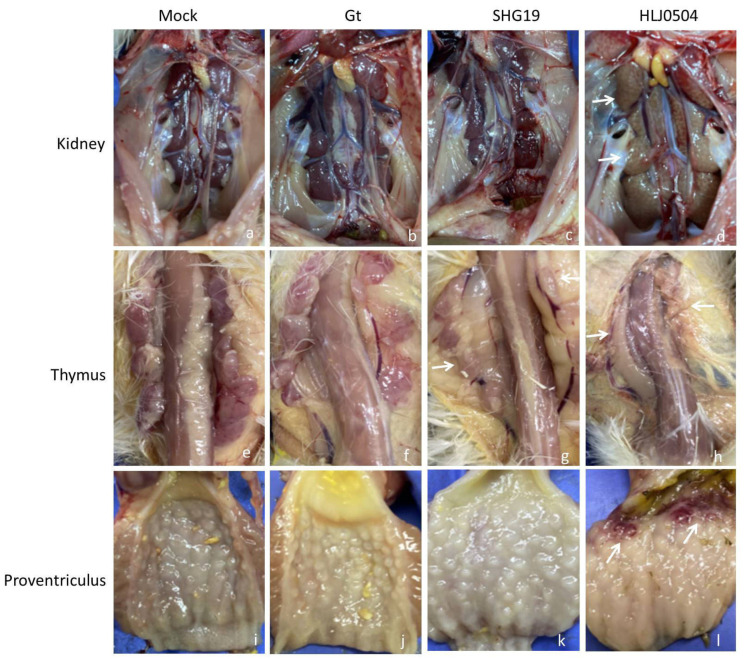
Gross lesions of the kidney (**a**–**d**), thymus (**e**–**h**), and proventriculus (**i**–**l**) of group HLJ0504, SHG19, Gt, and Mock. Pathological changes such as piebald in the kidney, thymus atrophy, and proventriculus hemorrhage were marked with arrows.

Thymus: Compared to the Mock group, the thymus of chickens in the HLJ0504 group was significantly atrophied, starting at 60 h p.i. Histopathological examination showed that lymphocytes were significantly reduced due to necrosis and that the macrophages over-proliferated. No significant lesions were found in the thymus of the chickens in the SHG19 and Gt groups (Figure 5).

Kidney: For the HLJ0504 group, 80% (4/5), 100% (3/3), and 66.7% (2/3) of the dissected chickens showed piebald in the kidneys at 4, 5, and 6 d p.i., respectively. The kidneys remained normal in the SHG19, Gt, and Mock groups (Figure 5).

Proventriculus: For the HLJ0504 group, 20% (1/5) to 33.3% (1/3) of dissected chickens presented proventriculus hemorrhage at 3, 4, 5, and 6 d p.i. The proventriculus bursas were normal in the SHG19, Gt, and Mock control groups (Figure 5).

### 3.5. EM Observation of the Viruses

**Figure 6:**
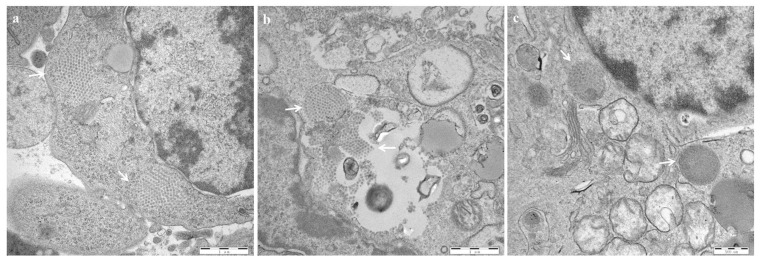
Electron microscopic observation of the infectious bursal disease virus (IBDV) in the bursa. (**a**) HLJ0504 group. (**b**) SHG19 group. (**c**) Gt group. The IBDV particles arranged in a lattice pattern were marked by a white arrow.

EM observations showed that virus particles arranged in a lattice pattern with a diameter of approximately 60 nm could be seen in the cytoplasm of the bursal tissues of both the HLJ0504 (Figure 6a) and SHG19 groups (Figure 6b) at 60 h p.i. For the Gt group, virus particles were not observed at 60 h, 72 h, and 5 d p.i.; relatively few IBDV were observed only at 14 d p.i. (Figure 6c).

### 3.6. Viral RNA Detection in Different Tissues

**Figure 7:**
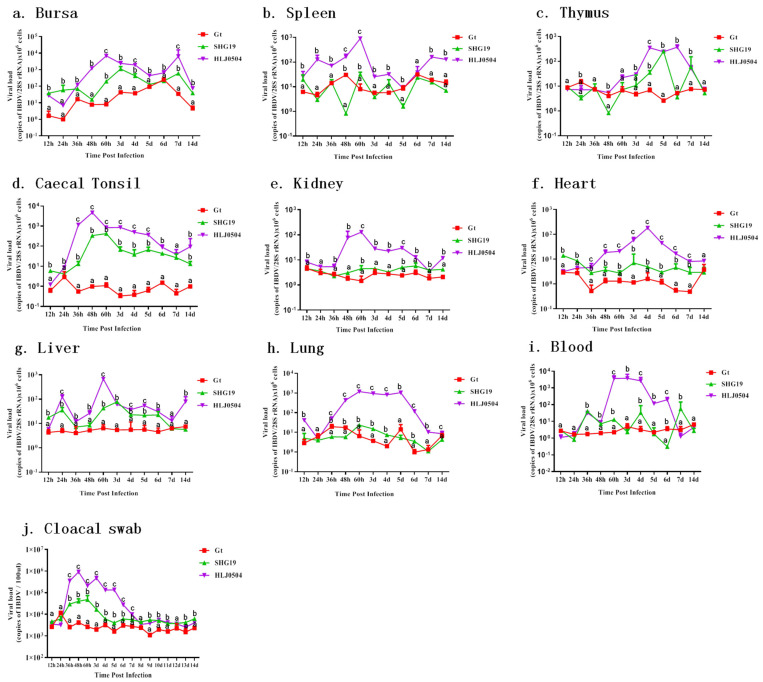
In vivo distribution and load of IBDV. (**a**) Bursa. (**b**) Spleen. (**c**) Thymus. (**d**) Caecal tonsil. (**e**) Kidney. (**f**) Heart. (**g**) Liver. (**h**) lung. (**i**) Blood. (**j**) Cloacal swab. The values for each group at each time point were averaged over three chickens, except for the HLJ0504 group at 14 d p.i. (*n* = 1). Treatments sharing different lowercase letter differ significantly at a confidence level (*p* < 0.05).

Major lymphoid tissues: In the bursa (Figure 7a), the HLJ0504 group had the highest viral load. The virus rapidly replicated after infection, and the viral load reached its maximum at 60 h p.i. (6879 ± 151 (copies of IBDV RNA/28S rRNA × 10^6^ cells)) and was maintained at a high level thereafter. Chickens in the SHG19 group had the second highest viral load with a delayed replication peak at 72 h p.i. (996 ± 120), which was approximately 5.9-fold lower than the maximum viral load in the HLJ0504 group. The viral load in the Gt group slowly increased, with a maximum value reached at 6 d p.i. (251 ± 2). In the spleen (Figure 7b), viral load was the highest in the HLJ0504 group, with a maximum viral load detected at 60 h p.i. (899 ± 16), while the viral loads of the spleen were low in both the SHG19 and Gt groups. In the thymus (Figure 7c), the viral load in the HLJ0504 group reached its maximum from 4 d p.i. to 6 d p.i. (mean, 326), and the viral load in the SHG19 group reached its maximum (256 ± 28) at 5 d p.i. The difference in viral load of the thymus between the Gt group and the Mock group was not significant. In the caecal tonsil (Figure 7d), the viral load in the HLJ0504 group increased rapidly after infection, reaching a high viral load of 4684 ± 774 at 48 h p.i. The SHG19 group reached its highest viral load between 48 h p.i. and 60 h p.i. (mean, 381). The difference in the viral load of the caecal tonsil was not significant between the Gt and Mock groups.

Non-lymphoid tissues: Viral load was also detected in non-lymphoid tissues such as the kidney (Figure 7e), heart (Figure 7f), liver (Figure 7g), and lung (Figure 7h) of the HLJ0504 group, with the viral load during 60 h p.i. to 5 d p.i also reaching approximately 1000 in the lungs. In the SHG19 group, the kidney, heart, liver, and lung had very low viral loads. Differences in viral loads in these non-lymphoid tissues between the Gt and Mock groups were not significant.

Blood: The HLJ0504 group also had a high viral load in the blood, with the average viral titers from 60 h p.i. to 4 d p.i. reaching 3.4 × 10^3^. The SHG19 group also had a detectable level of virus in the blood, but with lower titers. The overall difference between the Gt and Mock control groups was not significant (Figure 7i).

Cloacal swab: The HLJ0504 group had higher viral loads in the cloacal swab samples, with viral loads in all 36 h p.i. to 5 d p.i samples being above 1.3 × 10^5^ copies/100 μL, among which the viral load at 48 h p.i. reached 9.2 × 10^5^ copies/100 μL. The SHG19 group had viral loads with above 1.7 × 10^4^ copies/100 μL from 36 h p.i. to 3 d p.i., among which the viral load at 60 h p.i. reached 4.8 × 10^4^ copies/100 ul. The overall difference between the Gt group and the Mock group was not significant (Figure 7j).

### 3.7. Antibody Titers

**Figure 8:**
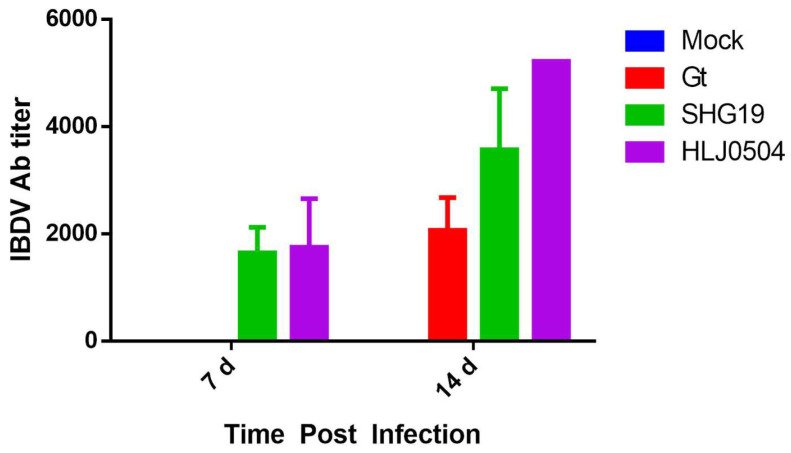
IBDV antibody titers in serum. The values for each group at each time point were averaged over five chickens, except for the HLJ0504 group at 7 d p.i. (*n* = 3) and 14 d p.i. (*n* = 1).

At 7 d p.i., IBDV antibodies were detected in sera of samples from the HLJ0504 and SHG19 groups with ELISA, and the concentrations of antibodies were significantly higher at 14 d p.i. In the Gt group, antibodies were not detected at 7 d p.i. but turned positive at 14 d p.i. At 14 d p.i., the highest mean antibody level was found in the HLJ0504 group, 1.5- and 2.5-fold higher than in the SHG19 and Gt groups, respectively (Figure 8).

### 3.8. Comparison of Expression Levels of Inflammation-Related Genes

**Figure 9:**
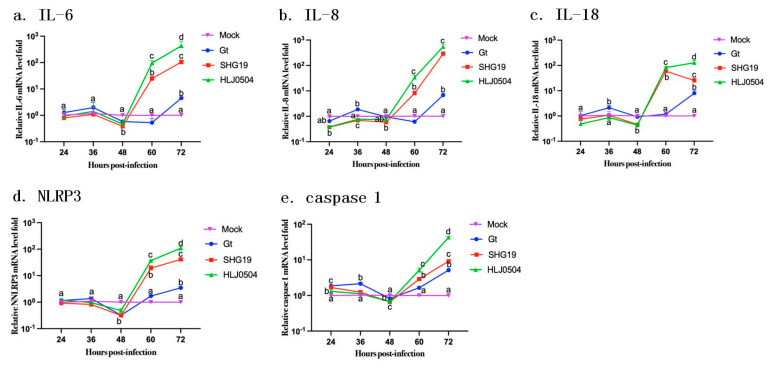
Inflammation-related genes expression in DT40 cells infected with different IBDV strains. The mRNA levels of IL-6 (**a**), IL-8 (**b**), IL-18 (**c**), NLRP3 (**d**), and caspase 1 (**e**) at 24, 36, 48, 60, and 72 h post-infection were detected by qRT-PCR. Treatments sharing different lowercase letter differ significantly at a confidence level (*p* < 0.05).

The expression levels of inflammation-related gene IL-6, IL-8, IL-18, NLRP3, and caspase-1 in the infected DT40 cells were showed in Figure 9. After 60 h p.i., the expression level of inflammatory factors triggered by the HLJ0504 was the highest, followed by the SHG19 group; except for 72 h p.i., there was no significant difference in inflammation-related genes expression between the Gt group and the mock control group.

## 4. Discussion

The continuous emergence of new strains of IBDV and the co-existence of multiple epidemic strains have been widely reported [7,12,16,20,21]. For example, in Italy, both a new genotype ITA strain and a classic IBDV induced sub-clinical damages [16]. Recently, the comparative pathogenicity of vvIBDV, classic IBDV, and reassortant IBDV circulating in Bangladesh was performed [21]. Systematically studying and comparing the pathogenic characteristics of these epidemic strains is of great significance for the comprehensive prevention and control of IBD.

The vvIBDV and nVarIBDV are the two current dominant strains in China [10,11,12], while the attIBDV strain is used as a vaccine strain to prevent the infection of vvIBDV, and nVarIBDV is featured with its ability of immune escape [9,22]. The pathogenicity of vvIBDV from China and Europe has been compared using SPF chickens, which showed that all the vvIBDVs studied including HLJ0504 strain could lead to high mortality and damage to the bursa [23]. It has been identified that nVarIBDV such as the SHG19 strain did not kill chickens but severely damaged immune organs [9,22]. The Gt strain has been confirmed as attenuated strain [3,24]. However, the replication kinetics of these IBDV in the target organ of bursa has not been detected, and the viral titer and the induced lesions of these IBDV in other non-target organs have not been systematically studied. This study not only confirmed the previous research results on the pathogenicity of these strains but also systematically compared the pathogenicity, tissue distribution, replication kinetics, and virus excretion of vvIBDV (HLJ0504), nVarIBDV (SHG19), and attIBDV (Gt).

The results of this study showed significant differences in clinical signs and tissue damage caused by IBDV strains with different virulence in infected chickens. The vvIBDV strain (HLJ0504) had the highest pathogenicity, the highest replication efficiency, the ability to cause significant damage to the bursa and other organ tissues, and high fatality. The virus was distributed in almost all organs, with the highest titers in the bursa, followed by the caecal tonsil, spleen, and thymus. High viral titers were also detected in the blood, with the 60 h p.i. to 4 d p.i. period being a period of severe viremia. EM observation showed that a large number of IBDV virion scattered in a lattice pattern were found in the infected bursa. Furthermore, analysis of the cloacal swabs revealed that the HLJ0504 group had significant virus excretion. The apparent signs, the reflection of in vivo lesions and viral proliferation, included the onset of depression in infected chickens from 48 h p.i., the peak period of death at 3–5 d p.i., and the final mortality of 80%. The cause of bursal atrophy is essentially the atrophy of the lymphoid follicles and the breakdown and necrosis of B lymphocytes. The vvIBDV has been shown to induce changes in cytokine levels within the bursa, promoting inflammation and disturbance of the tissue microenvironment, which is a strategy to reduce the activity of B lymphocytes that the virus uses to escape or suppress the immune responses of the host [25,26,27].

The high pathogenicity and fatality of vvIBDV have made it a major threat to the healthy development of the poultry industry for more than 30 years. Compared to vvIBDV, chickens infected with the newly emerged nVarIBDV (SHG19) did not show obvious clinical signs and had no death, as did attIBDV (Gt group). Although histopathological damage to the bursa in the SHG19 and HLJ0504 groups first appeared at 36 h p.i., the replication efficiency of SHG19 was significantly lower than that of HLJ0504. The detection of viral load showed that the virus content of nVarIBDV was lower than that of vvIBDV, but significantly higher than that of attIBDV in bursa and other tissues. However, like vvIBDV, nVarIBDV can cause serious damage to the central immune organ bursa and a large number of necrosis and disintegration of B lymphocytes, but the time lag of general lesions and atrophy of bursa in SHG19 group is 12 h compared to the HLJ0504 group. The specific mechanism for the lagged onset in the SHG19 group requires further in-depth investigation. The HLJ0504 group also exhibited lesions, including thymic atrophy, piebald in the kidneys, and hemorrhage in the proventriculus, which were not observed in the SHG19 group. Furthermore, the influences on the spleen in the SHG19 and HLJ0504 group were similar. Both vvIBDV and nVarIBDV caused persistent splenomegaly after 36 h p.i. The tissue distribution of SHG19 was also the highest in the bursa, followed by the caecal tonsil, spleen, and thymus, and was the lowest in the non-immune organs, including the kidney, liver, heart, and lung. However, nVarIBDV titers in all these organs were much lower than those of the organs of the HLJ0504 group without pronounced viremia. The analysis of cloacal swab showed that the SHG19 group also had significant virus excretion, but the absolute values were much lower than the HLJ0504 group and higher than the Gt group. The SHG19 group had a significantly lower in vivo replication level, shorter duration of the disease, and ameliorated lesions compared with the HLJ0504 group, but the virus was still able to replicate rapidly in the bursa and be excreted externally, which could be one reason the nVarIBDV did not kill chickens but became one of the dominant epidemic strains. 

The attenuated strain (Gt) was non-pathogenic, with slow replication and low titer. No significant clinical, autopsy, or pathological signs or lesions were observed in chickens inoculated with Gt, which was significantly different from vvIBDV and nVarIBDV. After Gt infection, low levels of viral replication were detectable only in the bursa, and only a small amount of clustered lattice-like IBDV particles were observed in the bursa at 14 d p.i, and there were no significant virus excretions from the infected chickens. The difference in the in vivo replication efficiency of the virus is an important influencer of the difference in the pathogenicity of different strains [28,29]. Furthermore, virus strains with different virulence could trigger different levels of immune response. The rate and titer of antibody production have been shown to be related to the degree of in vivo virus replication [30].

The elucidation of the underlying pathogenic mechanisms of viruses usually relies on in vitro susceptible cell lines. DT40 cell is the cell line derived from pre-B lymphocytes in the bursa of chicken [31]. Recently, as an in vitro infection model, DT40 cells were used for preliminary research on the biological characteristics of wild strains of IBDV [17]. Preliminarily, we compared the expression of inflammation-related genes in DT40 cells infected with different IBDV strains. The results suggest that infection with HLJ0504 and SHG19 can significantly up-regulate the expression of inflammatory host genes such as IL-6, IL-8, IL-18, NLRP3, and caspase 1, which may be the cause of histopathological changes such as inflammatory cell infiltration and inflammatory mucus exudation in bursa. This study mainly compared the pathogenic and lethal characteristics of three different virus strains using animal model, of which the molecular mechanisms involving in host inflammatory factors still needs further in-depth researches.

## 5. Conclusions

In this study, for the first time, the biological characteristics of vvIBDV, nVarIBDV, and attIBDV were studied comprehensively and comparatively using an SPF chickens infection model, providing insight into the deep understanding various pathogenicity mechanism among strains with different virulence. It is also important for developing an integrated prevention and control strategy for co-infection of various strains of IBDV.

## Figures and Tables

**Table 1:** RT-qPCR probes and primers for IBDV and inflammation-related genes.

Primers	Sequence (5′-3′)	5′Modification	3′Modification
28S-Probe	AGGACCGCTACGGACCTCCACCA	FAM	TAMRA
28S-F	GGCGAAGCCAGAGGAAACT	-	-
28S-R	GACGACCGATTTGCACGTC	-	-
IBDV-Probe	CGGCGTCCATTCCGGACGAC	FAM	BHQ-1
IBDV-VP5-F	GAGCCTTCTGATGCCAACAAC	-	-
IBDV-VP5-R	CAAATTGTAGGTCGAGGTCTCTGA	-	-
IL-6-F	GCCTGGAATTATCAAAGTAACCC	-	-
IL-6-R	CCTCTGCTGCCATTCCAC	-	-
IL-8-F	AAGATGTGAAGCTGACGCCAA	-	-
IL-8-R	TGGCCATAAGTGCCTTTACGA	-	-
IL-18-F	CCAGTTGCTTGTGGTTCGTC	-	-
IL-18-R	CGCTGAATGCAACAGGCATC	-	-
NLRP3-F	TAGAGTACGCGGGTGAAGGA	-	-
NLRP3-R	CTGTGAAACTGCCCAACACG	-	-
caspase-1-F	GGCAGTTGCCAACACAAGAG	-	-
caspase-1-R	CTCCCTGGCAAGAATTCGGT	-	-

## Data Availability

Data can be requested by writing to the author.

## References

[1] Trapp J., Rautenschlein S. (2022). Infectious Bursal Disease Virus’ Interferences with Host Immune Cells: What Do We Know?. Avian Pathol..

[2] Graziosi G., Catelli E., Fanelli A., Lupini C. (2022). Infectious Bursal Disease Virus in Free-Living Wild Birds: A Systematic Review and Meta-Analysis of Its Sero-Viroprevalence on a Global Scale. Transbound. Emerg. Dis..

[3] Wang X.M., Zeng X.W., Gao H.L., Fu C.Y., Wei P. (2004). Changes in VP2 Gene during the Attenuation of Very Virulent Infectious Bursal Disease Virus Strain Gx Isolated in China. Avian Dis..

[4] Fan L., Wu T., Hussain A., Gao Y., Zeng X., Wang Y., Gao L., Li K., Wang Y., Liu C. (2019). Novel Variant Strains of Infectious Bursal Disease Virus Isolated in China. Vet. Microbiol..

[5] Myint O., Suwanruengsri M., Araki K., Izzati U.Z., Pornthummawat A., Nueangphuet P., Fuke N., Hirai T., Jackwood D.J., Yamaguchi R. (2021). Bursa Atrophy at 28 Days Old Caused by Variant Infectious Bursal Disease Virus Has a Negative Economic Impact on Broiler Farms in Japan. Avian Pathol..

[6] Thai T.N., Jang I., Kim H.-A., Kim H.-S., Kwon Y.-K., Kim H.-R. (2021). Characterization of Antigenic Variant Infectious Bursal Disease Virus Strains Identified in South Korea. Avian Pathol..

[7] Aliyu H.B., Hair-Bejo M., Omar A.R., Ideris A. (2021). Genetic Diversity of Recent Infectious Bursal Disease Viruses Isolated From Vaccinated Poultry Flocks in Malaysia. Front. Vet. Sci..

[8] Wang Y., Jiang N., Fan L., Niu X., Zhang W., Huang M., Gao L., Li K., Gao Y., Liu C. (2021). Identification and Pathogenicity Evaluation of a Novel Reassortant Infectious Bursal Disease Virus (Genotype A2dB3). Viruses.

[9] Hou B., Wang C., Luo Z., Shao G. (2022). Commercial Vaccines Used in China Do Not Protect against a Novel Infectious Bursal Disease Virus Variant Isolated in Fujian. Vet. Rec..

[10] Fan L., Wang Y., Jiang N., Gao Y., Niu X., Zhang W., Huang M., Bao K., Liu A., Wang S. (2022). Residues 318 and 323 in Capsid Protein Are Involved in Immune Circumvention of the Atypical Epizootic Infection of Infectious Bursal Disease Virus. Front. Microbiol..

[11] Jiang N., Wang Y., Zhang W., Niu X., Huang M., Gao Y., Liu A., Gao L., Li K., Pan Q. (2021). Genotyping and Molecular Characterization of Infectious Bursal Disease Virus Identified in Important Poultry-Raising Areas of China During 2019 and 2020. Front.Vet. Sci..

[12] Zhang W., Wang X., Gao Y., Qi X. (2022). The Over-40-Years-Epidemic of Infectious Bursal Disease Virus in China. Viruses.

[13] Qi X., Gao L., Qin L., Deng X., Wu G., Zhang L., Yu F., Ren X., Gao Y., Gao H. (2011). Genomic Sequencing and Molecular Characteristics of a Very Virulent Strain of Infectious Bursal Disease Virus Isolated in China. Agric. Sci. Tech..

[14] Fan L., Wang Y., Jiang N., Gao L., Li K., Gao Y., Cui H., Pan Q., Liu C., Zhang Y. (2020). A Reassortment Vaccine Candidate of the Novel Variant Infectious Bursal Disease Virus. Vet. Microbiol..

[15] Nouën C.L., Toquin D., Müller H., Raue R., Kean K.M., Langlois P., Cherbonnel M., Eterradossi N. (2012). Different Domains of the RNA Polymerase of Infectious Bursal Disease Virus Contribute to Virulence. PLoS ONE.

[16] Lupini C., Felice V., Silveira F., Mescolini G., Berto G., Listorti V., Cecchinato M., Catelli E. (2020). Comparative in Vivo Pathogenicity Study of an ITA Genotype Isolate (G6) of Infectious Bursal Disease Virus. Transbound. Emerg. Dis..

[17] Liu A., Pan Q., Li Y., Yan N., Wang J., Yang B., Chen Z., Qi X., Gao Y., Gao L. (2020). Identification of Chicken CD74 as a Novel Cellular Attachment Receptor for Infectious Bursal Disease Virus in Bursa B Lymphocytes. J. Virol..

[18] Wang Y., Qi X., Gao H., Gao Y., Lin H., Song X., Pei L., Wang X. (2009). Comparative Study of the Replication of Infectious Bursal Disease Virus in DF-1 Cell Line and Chicken Embryo Fibroblasts Evaluated by a New Real-Time RT-PCR. J. Virol. Methods.

[19] Ragland W.L., Novak R., El-Attrache J., Savić V., Ester K. (2002). Chicken Anemia Virus and Infectious Bursal Disease Virus Interfere with Transcription of Chicken IFN-Alpha and IFN-Gamma mRNA. J. Interferon Cytokine Res..

[20] Legnardi M., Franzo G., Tucciarone C.M., Koutoulis K., Cecchinato M. (2023). Infectious Bursal Disease Virus in Western Europe: The Rise of Reassortant Strains as the Dominant Field Threat. Avian Pathol..

[21] Nooruzzaman M., Hossain I., Rahman M.M., Uddin A.J., Mustari A., Parvin R., Chowdhury E.H., Islam M.R. (2022). Comparative Pathogenicity of Infectious Bursal Disease Viruses of Three Different Genotypes. Microb. Pathog..

[22] Fan L., Wang Y., Jiang N., Chen M., Gao L., Li K., Gao Y., Cui H., Pan Q., Liu C. (2020). Novel Variant Infectious Bursal Disease Virus Suppresses Newcastle Disease Vaccination in Broiler and Layer Chickens. Poult. Sci..

[23] Li K., Courtillon C., Guionie O., Allée C., Amelot M., Qi X., Gao Y., Wang X., Eterradossi N. (2015). Genetic, Antigenic and Pathogenic Characterization of Four Infectious Bursal Disease Virus Isolates from China Suggests Continued Evolution of Very Virulent Viruses. Infec. Genet. Evol..

[24] Wang Y., Qi X., Kang Z., Yu F., Qin L., Gao H., Gao Y., Wang X. (2010). A Single Amino Acid in the C-Terminus of VP3 Protein Influences the Replication of Attenuated Infectious Bursal Disease Virus In Vitro and In Vivo. Antiviral. Res..

[25] Erickson K.L., Gershwin M.E., Abplanalp H., Ikeda R., Benedict A.A. (1982). Inherited 7S Immunoglobulin Deficiency of Chickens Is Associated with Bursal Degeneration Anomalies. Dev. Comp. Immunol..

[26] Huang X., Liu W., Zhang J., Liu Z., Wang M., Wang L., Zhou H., Jiang Y., Cui W., Qiao X. (2021). Very Virulent Infectious Bursal Disease Virus-Induced Immune Injury Is Involved in Inflammation, Apoptosis, and Inflammatory Cytokines Imbalance in the Bursa of Fabricius. Dev. Comp. Immunol..

[27] Pereira A.H., Vasconcelos A.L., Silva V.L., Nogueira B.S., Silva A.C., Pacheco R.C., Souza M.A., Colodel E.M., Ubiali D.G., Biondo A.W. (2022). Natural SARS-CoV-2 Infection in a Free-Ranging Black-Tailed Marmoset (Mico Melanurus) from an Urban Area in Mid-West Brazil. J. Comp. Pathol..

[28] Bassat Q., Varo R., Hurtado J.C., Marimon L., Ferrando M., Ismail M.R., Carrilho C., Fernandes F., Castro P., Maixenchs M. (2021). Minimally Invasive Tissue Sampling as an Alternative to Complete Diagnostic Autopsies in the Context of Epidemic Outbreaks and Pandemics: The Example of Coronavirus Disease 2019 (COVID-19). Clin. Infec. Dis..

[29] Takenaka-Uema A., Matsugo H., Ohira K., Sekine W., Murakami S., Horimoto T. (2022). Different Organ and Tissue Tropism between Akabane Virus Genogroups in a Mouse Model. Virus Res..

[30] Hamad M., Hassanin O., Ali F.A.Z., Ibrahim R.S., Abd-Elghaffar S.K., Saif-Edin M. (2020). Comparative Study on Dynamic and Immunopathology of Four Intermediate-plus Infectious Bursal Disease (IBD) Vaccines in Commercial Broiler Chickens. Vet. Res. Commun..

[31] Baba T.W., Giroir B.P., Humphries E.H. (1985). Cell Lines Derived from Avian Lymphomas Exhibit Two Distinct Phenotypes. Virology.

